# Innate immunology in COVID-19—a living review. Part I: viral entry, sensing and evasion

**DOI:** 10.1093/oxfimm/iqaa004

**Published:** 2020-12-08

**Authors:** Clarissa Coveney, Michel Tellier, Fangfang Lu, Shayda Maleki-Toyserkani, Ruth Jones, Valentina M T Bart, Ellie Pring, Aljawharah Alrubayyi, Felix C Richter, D Oliver Scourfield, Jan Rehwinkel, Patrícia R S Rodrigues, Luke C Davies, Ester Gea-Mallorquí, The Oxford-Cardiff COVID19 Literature Consortium

**Affiliations:** 1 Kennedy Institute of Rheumatology, University of Oxford, Oxford, UK; 2 Sir William Dunn School of Pathology, University of Oxford, Oxford, UK; 3 Systems Immunity Research Institute, Division of Infection and Immunity, School of Medicine, Cardiff University, Cardiff, UK; 4 Dementia Research Institute, Division of Infection and Immunity, School of Medicine, Cardiff University, Cardiff, UK; 5 Viral Immunology Unit, Nuffield Department of Medicine, University of Oxford, Oxford, UK; 6 Medical Research Council Human Immunology Unit, Radcliffe Department of Medicine, Medical Research Council Weatherall Institute of Molecular Medicine, University of Oxford, Oxford, UK

**Keywords:** SARS-CoV-2, COVID-19, viral entry, viral evasion, host response, interferon

## Abstract

The coronavirus infectious disease 2019 (COVID-19) pandemic caused by severe acute respiratory syndrome coronavirus 2 (SARS-CoV-2) remains a world health concern and can cause severe disease and high mortality in susceptible groups. While vaccines offer a chance to treat disease, prophylactic and anti-viral treatments are still of vital importance, especially in context of the mutative ability of this group of viruses. Therefore, it is essential to elucidate the molecular mechanisms of viral entry, innate sensing and immune evasion of SARS-CoV-2, which control the triggers of the subsequent excessive inflammatory response. Viral evasion strategies directly target anti-viral immunity, counteracting host restriction factors and hijacking signalling pathways to interfere with interferon production. In Part I of this review, we examine SARS-CoV-2 viral entry and the described immune evasion mechanisms to provide a perspective on how the failure in initial viral sensing by infected cells can lead to immune dysregulation causing fatal COVID-19, discussed in Part II.

## Graphical Abstract

**Graphical Abstract:**
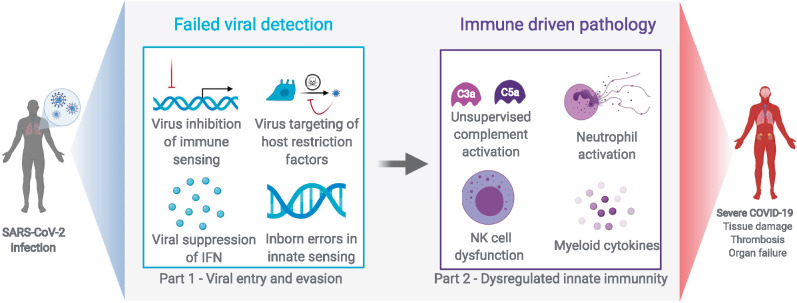

**Box 1: Why does your reviewed topic matter in the pandemic?**

The Coronavirus disease 2019 (COVID-19) pandemic is caused by a novel virus termed severe acute respiratory syndrome coronavirus 2 (SARS-CoV-2). Viral entry into host cells is the first stage of infection and a promising drug target. After viral entry, viral sensing by the host cell is essential for the innate immune response to be triggered. However, SARS-CoV-2 can evade the host immune response by targeting cellular host factors. Therefore, it is pivotal to elucidate the mechanisms of viral entry, innate sensing and immune evasion of SARS-CoV-2, which can provide a basis for therapeutic development.

**Box 2: What is the consensus?**

To initiate infection, the spike (S) protein of SARS-CoV-2 engages the host cell receptor, angiotensin-converting enzyme II (ACE2), which is highly expressed in lung tissues. Viral materials released into the cytosol can be sensed by the host cell viral recognition machinery which subsequently activates the anti-viral interferon (IFN) pathway. However, SARS-CoV-2 encodes for proteins that counteract these viral recognition pathways or function as IFN antagonists, leading to reduced IFN signalling. In addition, subsets of patients present with genetic mutations in the TLR3 and IRF7 signalling pathways, which lead to defective IFN responses and a worse outcome. This impaired sensing of virus may allow viral replication, which leads to cell damage and systematic immune dysregulation.

## INTRODUCTION

The coronavirus infectious disease 2019 (COVID-19) pandemic has had devastating global impacts on human health and the economy. Caused by a novel virus termed severe acute respiratory syndrome coronavirus 2 (SARS-CoV-2), COVID-19 is heterogeneous in clinical presentation and outcome: it is estimated that 20% of patients are asymptomatic, most progress with mild infection and a minority experience acute respiratory distress syndrome [1] that can be fatal. While long-term health implications of SARS-CoV-2 infection are unknown, acute symptoms can cause respiratory, intestinal, kidney and neurological complications as well as loss of smell and taste [2–5]. Disease severity is correlated with age, ethnicity and underlying health conditions including diabetes, obesity and heart disease. It is unclear why some patients develop severe disease, though lack of proper viral recognition and a dysregulated immune response are involved in pathogenicity. After entry into the host cell, viral pathogens have evolved strategies to prevent triggering of anti-viral immune responses by counteracting host anti-viral machinery and pathogen recognition. Innate immune cells are the primary responders to viral infections and key to elicit specific adaptive and memory immune responses through cytokine secretion and antigen presentation [6]. In severe COVID-19, a major dysregulation of innate responses with excessive cytokine production triggers widespread systemic inflammation exacerbating disease [6]. In this review (Part I), we examine recent research elucidating the mechanisms of SARS-CoV-2 cell entry, viral sensing and immune evasion. In Part II, we will discuss the responses of innate immune cells including natural killer (NK) cells, macrophages, monocytes and neutrophils to SARS-CoV-2 infection, including their role in mediating antiviral responses and their contribution to disease pathogenesis. Finally, we highlight promising targets for therapeutic interventions.

### Viral entry

Viral entry into a host cell usually requires proteins in the viral envelope engaging with cell surface receptors. In COVID-19, SARS-CoV-2 spike (S) glycoprotein binds to the angiotensin-converting enzyme II (ACE2) [7] receptor with high affinity [8, 9], mediating viral entry. ACE2 is a carboxypeptidase and an important component of the renin–angiotensin system (RAS), a system of regulatory mediators of blood pressure and organ homeostasis involved in the regulation of inflammatory pathways in the lung [10]. ACE2 is highly expressed in endothelial and epithelial cells of the heart, kidney, cornea, liver, gut and airways [11]. In the airways, multi-ciliated respiratory tract cells play an important role as a first line of defence through mucociliary pathogen clearance. Ciliated epithelia cells express high levels of ACE2 mRNA [11, 12] and SARS-CoV-2 infection results in loss of motile cilia, impacting airway immunity [13]. ACE2 expression varies by age and ethnicity and has been associated with comorbidities and severe COVID-19 [14–16]. This can be explained by the protective role of ACE2 in RAS of tissues severely affected by COVID-19 [17]. Additionally, factors that maintain ACE2 expression are important, such as HMGB1, which was demonstrated to be crucial for viral entry [18]. Potential prophylactic treatments to block viral entry by impairing ACE2-S interaction are listed in Table 1.

**Table 1:** Potential treatments

Therapy	Target	Mechanism	References
Monoclonal antibody	ACE2	Block the interaction between SARS-CoV-2 S protein and ACE2.	Chen *et al.* [19]
Angiotensive enzyme (ACE) inhibitor and angiotensin receptor blocker (ARB) drugs	ACE, ACE2, angiotensin receptor	Inhibition of ACE activity or blockage of the angiotensin receptor activity.	Hippisley-Cox *et al.* [20]
Type I IFN supplementation	IFN-β, IFN-α2b, IFN-α1b	Severe COVID-19 patients have shown reduced type I IFN responses. Type I IFN supplementation reduced the duration of inflammatory markers in mild disease and prevented COVID-19 infection in highly exposed individuals.	Hung *et al.* [21] Zhou *et al.* [22] Meng *et al.* [23] (prevented infection in highly exposed individuals)
Paricalcitol	ADAM17/ACE2	Regulates ACE2 shedding through ADAM17.	Riera *et al.* [24]
Camosat mesyalte	TMPRSS2	Inhibitor of protease	Hoffmann *et al.* [7]
Decanoyl-RVKR-chloromethylketone, Naphthofluorescein	Furin	Inhibitor of protease	Cheng *et al.* [25]
Chloroquine, hydroxychloroquine, chlorpromazine	Endocytic pathway	Block the fusion of viral membrane and the endosomal/lysosomal membrane	Chen *et al.* [26] Plaze *et al.* [27]
Remdesivir, Enisamium	SARS-CoV-2 RNA polymerase	Inhibition of the RNA-dependent, RNA polymerase.	Beigel *et al.* [28] Walker *et al.* [29]

Following receptor engagement, fusion of SARS-CoV-2 with the host cell requires transmembrane serine protease 2 (TMPRSS2) to cleave the S protein at the S1/S2 cleavage site [7]. S1 mediates receptor binding, while S2 is required for membrane fusion; both are needed for endocytosis [30] into the host cell via dynamin/clathrin machinery [31]. Additionally, SARS-CoV-2 S protein can be cleaved and primed by the protease furin between its S1/S2 domains [32–34]. The dominant mutant D614G, with a single amino acid mutation in the SARS-CoV-2 S protein, has been linked to increased infectivity, possibly via reduced shedding of the S1 domain [35, 36]. S1 also requires binding of Neuropilin-1 (NRP1) for the viral entry process and blocking this interaction reduced SARS-CoV-2 cell entry [37]. Alternatively, SARS-CoV-2 can enter through the late endosome/lysosome pathway, wherein the cellular cathepsin L proteinase cleaves the S protein and initiates fusion of viral and endosomal/lysosomal membranes [30]. An additional mediator of SARS-CoV-2 infection is A Disintegrin And Metalloproteinase 17 (ADAM17), which cleaves ACE2, thus shedding the receptor and reducing viral uptake in cells [38–40]. Drugs impairing endocytic routes, targeting these proteases and the ADAM17/ACE2 axis are listed in Table 1.

Viral tropism determines the target cell of the virus. Therefore, it is important to note other putative receptors for SARS-CoV-2 entry including kidney injury molecule-1 (KIM-1—highly expressed in kidney cells) [41], CD147 [42], CD4 [43] and CD26 [44]. More work is needed to confirm these, although it is already known that anti-KIM-1 IgG and a KIM-1 inhibitor (TW-37) impaired SARS-CoV-2 endocytosis in human alveolar basal epithelial cells [41, 45]. It is still debated whether SARS-CoV-2 replicates in innate immune cells such as monocytes and macrophages which express ACE2, TMPRSS2 and ADAM17 [46] but SARS-CoV-2 infection did not induce a cytopathic effect, suggesting abortive infection in these cells [47].

### Innate sensing

Following virus entry, the viral genome is exposed, and viral proteins are synthesized by the host cell machinery for assembly of new virions. However, host cells can recognize this viral material (pathogen-associated molecular patterns—PAMPs) via pattern recognition receptors (PRRs). PRRs initiate signalling cascades culminating in the production of pro-inflammatory cytokines and IFNs, which upregulate IFN-stimulated genes (ISGs) that further direct innate and adaptive immunity.

PRRs include cytosolic RNA sensors such as retinoic acid-inducible Gene I (RIG-I) and melanoma differentiation gene 5 (MDA-5), while cytosolic DNA triggers the cyclic GMP–AMP synthase/stimulator of IFN genes (cGAS-STING) pathway. Indeed, *in vitro* infection with SARS-CoV-2 upregulated pathways for RIG-I signalling in Huh7 cells (liver cell line) [48] and activated MDA-5 in primary human epithelia and cell lines, inducing a robust type I and type III IFN response, though this induction failed to control viral replication [49]. SARS-CoV-2 also induces a cGAS-STING mediated NF-kB activation of inflammatory immune responses [50] and polymorphisms in STING have been suggested to produce a delayed over-secretion of IFN-β in SARS-CoV-2 infection [51].

Endosomal toll-like receptor 7 (TLR7) recognizes single-stranded viral RNA (ssRNA) and TLR3 binds double-stranded RNA (dsRNA) generated during viral replication. Therefore, inborn genetic errors affecting the TLR3 signalling pathway result in a defective type I IFN response and are associated with life-threatening COVID-19 [52]. Sex differences in innate immune sensing can also explain epidemiology underlying COVID-19 severity, as TLR genes are encoded on the X chromosome, causing higher expression and stronger innate immune activation in women [53]. Sex differences in COVID-19 have been evaluated extensively by Takashaki *et al.* who found that females produced more IFNα2 and that elevated innate cytokines correlated with disease progression, but only in females [54].

Fine tuning of IFN responses appears to be key for COVID-19 outcome, as shown by dysregulated responses attributed to auto-antibodies against IFN found in 10% of severe patients [55]. While IFNs are usually the primary anti-viral cytokines produced by cells sensing respiratory viruses, SARS-CoV-2 infection has been shown to elicit a dampened IFN-I and IFN-III response in human alveolar cells (A549) and ferrets [56]. The IFN response may not be absent but rather delayed, as shown in *in vitro* infection of lung Calu-3 cells [56], especially when compared to the respiratory Sendai virus [57]. Similarly, in infected human lung tissues, SARS-CoV-2 did not significantly induce types I, II or III IFNs [58]. These lower levels of IFN can be explained by effective viral immune evasion mechanisms. Therefore, early enhancement of IFN signalling may offer therapeutic benefit, and trials of IFN supplementation are listed in Table 1.

Other innate activation pathways involved in SARS-CoV-2 sensing include NOD-like receptor (NLR) activation and tumor necrosis factor (TNF)-α production [48]. NLR family pyrin domain-containing 3 (NLPR3) inflammasome activation by SARS-CoV-2 ORF3a was predicted based on SARS-CoV studies [59, 60] and has now been demonstrated via both ASC-dependent and ASC-independent pathways [61].

### Immune evasion

Viruses have evolved mechanisms to evade the activation of host innate immune responses [62]. For example, SARS-CoV-2 displays a range of molecules directly targeting the type I IFN pathway: ORF6 protein has been reported to inhibit both Type I IFN production and downstream signalling, the C-terminus region being critical for this antagonistic effect [57]. ORF6 has been shown to localize in the nuclear pore complex and block nuclear translocation for pSTAT1 [63] and IFN responsive factor (IRF) 3 [64], impairing IFN signalling. The IFN response was also found to be attenuated and linked to viral suppression of STAT1 phosphorylation in monocyte-derived macrophages and dendritic cells [65]. Studies using Sendai virus to mimic IFN response to SARS-CoV-2 revealed that together with ORF6, ORF8 and Nucleocapsid (N) contribute to the inhibition of the type I IFN response [66] and subsequently the NF-κB-responsive promoter via IFN-stimulated response element (ISRE) [67]. SARS-CoV-2 ORF3b is truncated and suppressed IFN induction more than the SARS-CoV variant when using Sendai virus [68]. Furthermore, SARS-CoV-2 PLpro cleaves ISG15 from IRF3, dampening the IFN response [69]. Non-structural proteins (NSP) also perform as IFN antagonists: SARS-CoV-2 NSP6 suppressed IRF3 phosphorylation through binding TANK binding kinase (TBK1), while NSP13 blocked TBK1 phosphorylation [64]. Screening SARS-CoV-2 proteins, Lei *et al.* found that NSP1, NSP3, NSP12, NSP13, NSP14, but also ORF3, ORF6 and structural M protein could inhibit the activation of the IFN-ß promoter after infection with Sendai virus [57]. SARS-CoV-2 NSP13, NSP14 and NSP15 can also act as IFN antagonists [66] but the mechanisms are still unclear. Interestingly, NSP2 and S protein activate IFN [57]; however, subsequent viral activity perhaps dampens this response. SARS-CoV-2 is also likely to share other evasion mechanisms with SARS and MERS, which have been extensively discussed elsewhere [65, 70–73].

Viral proteins also target cellular intrinsic mechanisms of defence, such as anti-viral host restriction factors: proteins that interfere with the viral life cycle. The C-terminus of SARS-CoV-2 NSP1 obstructs the mRNA entry tunnel of the 40S ribosomal subunit, resulting in translation shutoff of host mRNAs [74]. Other viral proteins including NSP5, NSP8, NSP13, N and envelope protein E interact with host factors involved in epigenetic and RNA regulation, which could interfere with the host response [75]. For instance, NSP16 inhibits pre-mRNA splicing [76], while NSP8 and NSP9 bind to the 7SL RNA component of the signal recognition particle (SRP) complex, interfering with protein trafficking to the cell membrane [76]. Martin-Sancho *et al*. extensively screened for ISGs acting as host restriction factors in the context of SARS-CoV-2 infection [77]. These ISGs include endosomal factors inhibiting viral entry, nucleic acid binding proteins, inhibitors of viral translation, regulators of membrane lipids and vesicle transport. For example, tetherin (BST2) binds newly synthesized viruses to the plasma membrane impairing viral release. SARS-CoV-2 ORF7a was shown to counteract tetherin to allow viral release [77]. SARS-CoV-2 ORF8 has also been suggested to downregulate surface MHC-I [78] by targeting it for lysosomal degradation, which would impact the function of NK cells and CD8 T-cells.

## Conclusion

**Figure 1:**
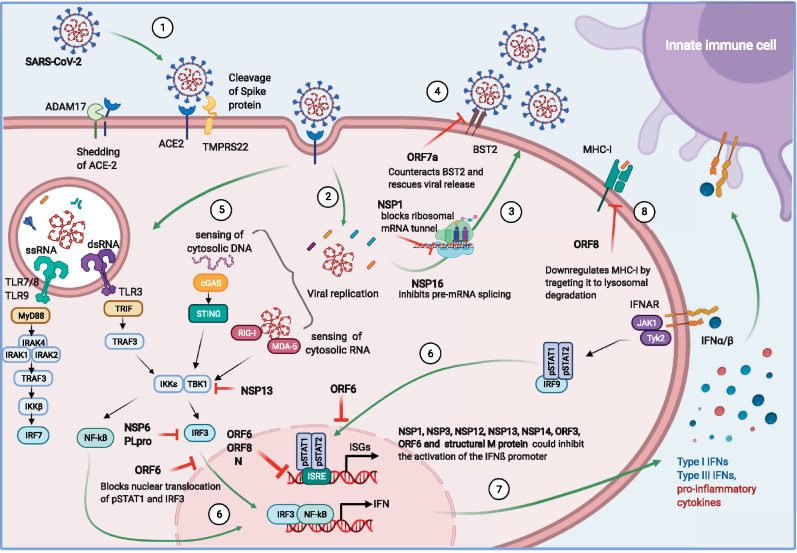
Viral entry and immune evasion. Graphical depiction of the reported SARS-CoV-2 mechanisms of immune detection and viral evasion in host cells. (1) Virus engagement to ACE2 and fusion to the cell membrane, (2) viral genetic material release into the cytoplasm, (3) late phase of the viral cycle: viral replication and release, (4) host restriction factors interfering with the viral life cycle: BST2 impeding viral release, (5) host immune sensing of viral components, (6) nuclear translocation of different regulatory factors that trigger transcriptional programs for pro-inflammatory cytokines, ISGs and IFN; and their release (7). (8) MHC-I antigenic presentation; and corresponding viral counteraction potentially affecting induction of immune responses.

The mechanisms of SARS-CoV-2 immune evasion at the early stages of infection are shown in Figure 1 and this understanding is a key for generating effective treatments against COVID-19, such as those listed in Table 1. The immune dysregulation causing fatal COVID-19 likely starts at the cellular level and identifying pathways involved in defective or altered responses is crucial to dampen the pathogenicity of the infection. As with most viruses, SARS-CoV-2 encodes numerous proteins that counteract host restriction factors or act as IFN antagonists, impairing recognition of the virus which allows for viral replication in the absence of antiviral immunity. SARS-CoV-2 has evolved diverse ways to evade innate mechanisms, which may facilitate high transmissibility and virulence. However, this evasion of the immune system cannot explain why only a small minority of patients progress to severe disease. Thus, other factors such as inborn genetic errors in sensing, existing autoantibodies, immunogenic tolerance and the immune response itself must also influence the outcomes of COVID-19. In Part II of this review, we investigate the consequences of failed immune sensing on the innate immune system, which results in immunopathology.
